# Systematic review and meta-analysis of the effectiveness of non-pharmacological interventions in restoring lower limb motor function after stroke

**DOI:** 10.3389/fnhum.2026.1864828

**Published:** 2026-09-25

**Authors:** Tokzhan Kispayeva, Inna Bolshakova, Akmaral Izbasarova, Zilola Mavlyanova, Tomiris Zhunussova

**Affiliations:** 1 School of Nursing Education, Karaganda Medical University, Karaganda, Kazakhstan; 2 Institute of Life Sciences, Karaganda Medical University, Karaganda, Kazakhstan; 3 Department of Physical Medicine and Rehabilitation and Sports Medicine, Asfendiyarov Kazakh National Medical University, Almaty, Kazakhstan; 4 Department of Medical Rehabilitation, Sports Medicine and Traditional Medicine, Samarkand State Medical University, Samarkand, Uzbekistan; 5 Karaganda Medical University, Karaganda, Kazakhstan

**Keywords:** Fugl–Meyer assessment, gait recovery, lower limb, meta-analysis, rehabilitation, robotic rehabilitation, stroke

## Abstract

**Objective:**

To evaluate the effectiveness of non-pharmacological interventions in improving lower limb motor function in patients after stroke based on a systematic review and meta-analysis of randomized controlled trials.

**Methods:**

A systematic literature search was conducted in PubMed (MEDLINE), Scopus, and the Cochrane Library, supplemented by manual screening of reference lists. Randomized controlled trials involving adult patients after stroke comparing non-pharmacological interventions with conventional rehabilitation were included. The primary outcome was lower limb motor function assessed by the Fugl–Meyer Assessment of the Lower Extremity (FMA-LE). The review was conducted in accordance with the PRISMA 2020 guidelines. Risk of bias was assessed using the Cochrane Risk of Bias 2 (RoB 2) tool. A random-effects model was applied to calculate pooled mean differences (MDs) with 95% confidence intervals.

**Results:**

Fifteen randomized controlled trials were included in the systematic review, of which 14 studies involving 623 participants were included in the quantitative synthesis. Non-pharmacological interventions demonstrated a statistically significant improvement in lower limb motor function compared with conventional rehabilitation (MD = 3.06, 95% CI 1.97–4.16; *Z* = 5.48, *p* < 0.00001), with substantial heterogeneity (*I*^2^ = 60%). In the subgroup analysis, robotic/mechanized rehabilitation interventions showed a significant positive effect (MD = 3.00, 95% CI 1.66–4.33; *I*^2^ = 57%), as did other non-pharmacological interventions (MD = 3.15, 95% CI 0.94–5.35; *I*^2^ = 68%). There was no statistically significant difference between the subgroups (*p* = 0.91; *I*^2^ = 0%).

**Conclusion:**

Non-pharmacological rehabilitation interventions were associated with a statistically significant improvement in lower limb motor function after stroke compared with conventional rehabilitation. Substantial heterogeneity was observed across the included studies, highlighting the need for further high-quality randomized controlled trials with standardized intervention protocols and longer follow-up.

**Systematic review registration:**

https://www.crd.york.ac.uk/PROSPERO/view/CRD420251154919.

## 1 Introduction

Stroke remains one of the leading causes of mortality and long-term disability worldwide (Feigin et al., 2024). Despite advances in acute medical care and improved survival rates, the prevalence of post-stroke disability remains high, placing a substantial burden on healthcare systems and society (GBD 2021 Stroke Collaborators, 2023; Cramer, 2020). A significant proportion of stroke survivors experience persistent functional limitations due to motor impairments, which reduce independence in daily activities and hinder social reintegration (Gittins et al., 2021).

Restoration of motor function is a central goal of post-stroke rehabilitation. Among post-stroke deficits, impairments of lower limb motor function are particularly important and include reduced muscle strength, impaired coordination, spasticity, and gait instability (Awad et al., 2020). Recent rehabilitation studies have also emphasized targeted approaches to restoring motor function after stroke (Kispaeva et al., 2025).

These impairments substantially limit functional independence, increase the risk of falls and negatively affect quality of life (Batchelor et al., 2021).

Current rehabilitation approaches encompass a wide range of methods aimed at improving motor function and functional performance. Conventional therapies, including therapeutic exercise, physiotherapy, and occupational therapy, remain the cornerstone of rehabilitation programs (Pollock et al., 2014). In recent years, technology-assisted rehabilitation methods have been increasingly introduced to enhance training intensity, standardization, and reproducibility (Mehrholz et al., 2020).

These approaches include robotic gait training systems, exoskeletons, and mechanized treadmill-based interventions with body-weight support (Louie and Eng, 2021; Hatem et al., 2016). Such technologies are hypothesized to facilitate neuroplasticity and promote more effective motor recovery after stroke (Morone et al., 2017). However, evidence from clinical trials evaluating these interventions remains heterogeneous, limiting the development of consistent clinical recommendations (Morone et al., 2017).

The Fugl–Meyer Assessment for Lower Extremity (FMA-LE) is one of the most widely validated tools for the quantitative evaluation of motor recovery after stroke (Gladstone et al., 2020; Sullivan et al., 2017). The use of a standardized outcome measure enables reliable data synthesis and facilitates a more accurate assessment of intervention effectiveness.

Therefore, a systematic review and meta-analysis of randomized controlled trials is warranted to evaluate the effectiveness of non-pharmacological interventions in improving lower limb motor function after stroke. The aim of this study was to quantitatively assess the effectiveness of rehabilitation technologies in restoring lower limb motor function in patients after stroke using the FMA-LE scale.

The novelty of the present study lies in the quantitative synthesis of randomized controlled trial data using a standardized clinical outcome measure, which allows for a more objective evaluation of the effectiveness of modern rehabilitation technologies after stroke.

## 2 Methods

This study was conducted as a systematic review and meta-analysis of randomized controlled trials in accordance with the PRISMA 2020 (Preferred Reporting Items for Systematic Reviews and Meta-Analyses) guidelines (Page et al., 2020). The study protocol was prospectively registered in the PROSPERO database (CRD420251154919) (PROSPERO, 2025).

### 2.1 Information sources and search strategy

A systematic literature search was conducted in three electronic databases: PubMed (MEDLINE), Scopus, and the Cochrane Library. To minimize the risk of missing relevant studies, manual screening of reference lists from included articles and previously published systematic reviews was also conducted. The search strategy combined keywords and Medical Subject Headings (MeSH) terms related to the population (stroke), lower limb motor function and gait, rehabilitation interventions, and randomized controlled trials. The search was limited to studies published between 2015 and 2025, involving adult participants (≥18 years) and human subjects. An example search query used in PubMed was: (“stroke” OR “cerebrovascular accident”) AND (“lower limb” OR “lower extremity” OR gait OR walking) AND (rehabilitation OR neurorehabilitation OR physiotherapy OR “physical therapy”) AND (“Fugl-Meyer” OR “Fugl Meyer” OR “FMA-LE” OR “Fugl-Meyer Assessment”).

### 2.2 Eligibility criteria

Eligibility criteria were defined according to the PICO framework. Randomized controlled trials involving adult patients (≥18 years) after stroke with impaired lower limb motor function were included, regardless of the stage of recovery. The interventions of interest included non-pharmacological rehabilitation approaches aimed at improving lower limb motor function, including robotic and mechanized rehabilitation, exoskeleton-assisted therapy, treadmill-based training, hydrotherapy, neurostimulation, repetitive transcranial magnetic stimulation, and balance-oriented interventions. Comparator groups included conventional rehabilitation, standard care, or an active control intervention. The primary outcome was lower limb motor function assessed using the Fugl–Meyer Assessment for the Lower Extremity (FMA-LE).

Studies were excluded from the systematic review if they were not randomized controlled trials, involved participants under 18 years of age, or did not report outcomes relevant to lower limb motor function. Studies were excluded from the quantitative synthesis if they did not report quantitative post-intervention FMA-LE data suitable for pooling.

### 2.3 Study selection

Study selection was conducted in two stages. First, titles and abstracts were screened to exclude clearly irrelevant studies. Second, full-text articles were assessed for eligibility according to the inclusion criteria. Screening and full-text assessment were performed independently by two reviewers (I. B. and T. K.). Disagreements were resolved through discussion with a third reviewer (A. I.). All records were imported into Zotero reference management software, and duplicates were removed.

### 2.4 Data extraction

The following data were extracted from each included study: author and year of publication, country, sample size of intervention and control groups, characteristics of interventions and control treatments, duration of the rehabilitation program, and lower limb motor function outcomes measured by FMA-LE (mean values and standard deviations). Where multiple eligible outcome measures or assessment time points were reported, post-intervention FMA-LE data were selected for the primary quantitative synthesis.

### 2.5 Risk of bias assessment

The risk of bias in the randomized controlled trials included in the quantitative synthesis was assessed using the Cochrane Risk of Bias 2 (RoB 2) tool, which evaluates five domains: randomization process, deviations from intended interventions, missing outcome data, measurement of outcomes, and selection of the reported result (Sterne et al., 2019). The assessment was performed independently by two reviewers (I. B. and T. K.), with disagreements resolved through discussion involving a third reviewer (A. I.).

### 2.6 Statistical analysis

Statistical analyses were performed using Review Manager (RevMan), version 5.4 (Cochrane Collaboration). The primary outcome was lower limb motor function measured by the Fugl–Meyer Assessment for the Lower Extremity (FMA-LE). Because all included studies assessed the same outcome using the same FMA-LE scale, mean differences (MDs) with 95% confidence intervals were calculated. A random-effects model was applied to account for expected clinical and methodological variability among studies.

Statistical heterogeneity was assessed using Cochran’s Q test and the *I*^2^ statistic. *I*^2^ values were interpreted as low (≤25%), moderate (25–50%), substantial (50–75%), and high (>75%) heterogeneity. Between-study variance (*τ*^2^) was estimated using the DerSimonian–Laird method.

A subgroup analysis was performed according to intervention type, comparing robotic and mechanized rehabilitation technologies with other non-pharmacological rehabilitation approaches. A leave-one-out sensitivity analysis was performed by sequentially excluding each study from the meta-analysis to assess the robustness of the pooled estimate.

### 2.7 Certainty of evidence

The certainty of evidence for the primary outcome (FMA-LE) was evaluated using the GRADE (Grading of Recommendations Assessment, Development and Evaluation) approach (Guyatt et al., 2008). The following domains were assessed: risk of bias, inconsistency, indirectness, and imprecision.

Since all included studies were randomized controlled trials, the initial level of evidence was considered high and was subsequently downgraded based on identified limitations.

## 3 Results

### 3.1 Study selection

The literature search identified records from PubMed (MEDLINE), Scopus, and the Cochrane Library. Following the screening and full-text eligibility assessment, 15 randomized controlled trials met the predefined inclusion criteria and were included in the systematic review. Fourteen studies involving 623 participants were included in the quantitative synthesis. One eligible study by Park et al. (2021), involving 20 participants, was retained for qualitative synthesis but was not included in the meta-analysis because it reported FMA-LE synergy subscores rather than the prespecified total FMA-LE score. The study selection process is presented in Figure 1.

**Figure 1:**
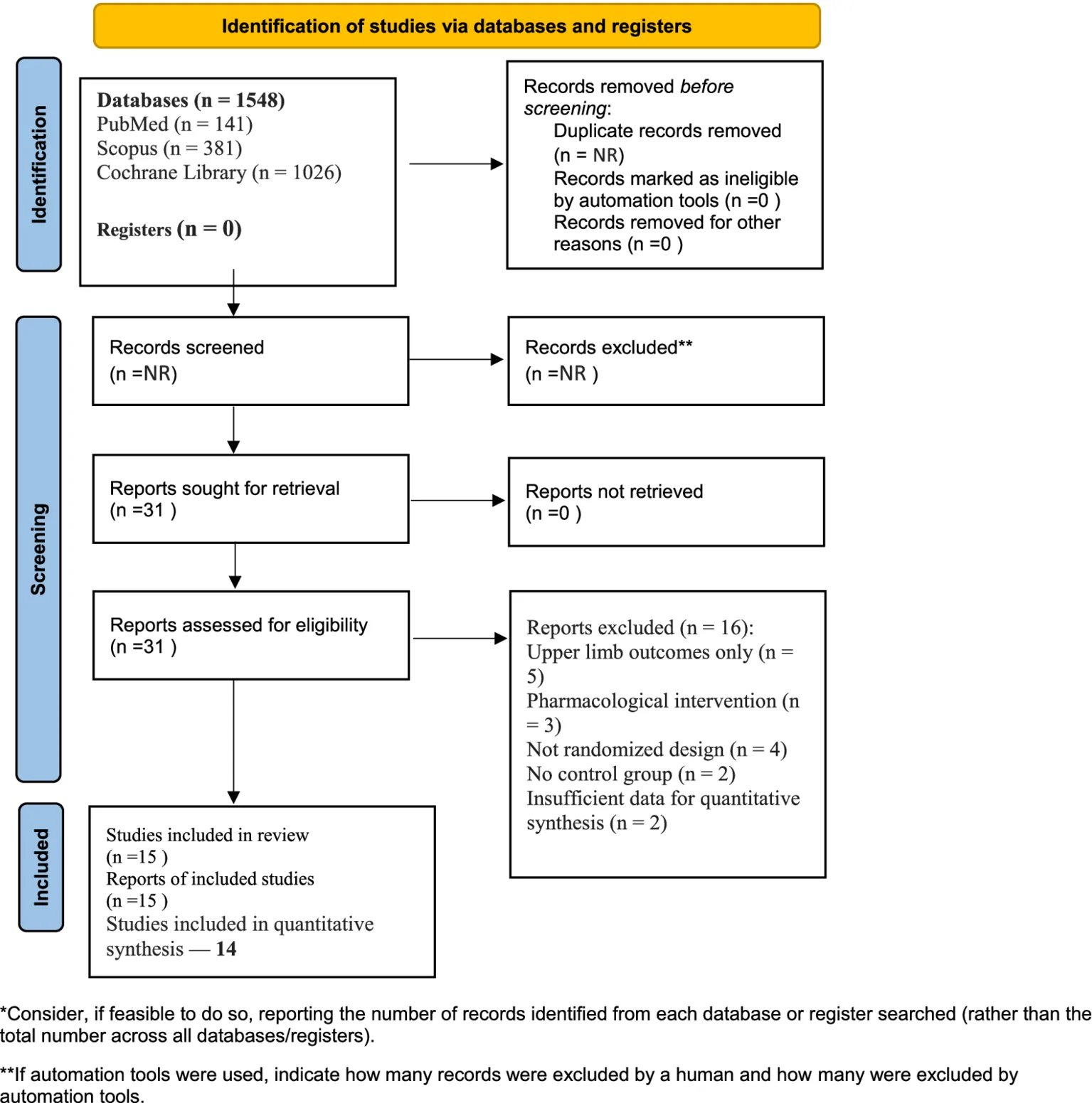
PRISMA 2020 flow diagram of the study selection process. The diagram presents the number of records identified, screened, excluded, and included, along with reasons for exclusion at the full-text stage. Source: Page et al. (2021). This work is licensed under CC BY 4.0. To view a copy of this license, visit https://creativecommons.org/licenses/by/4.0/.

### 3.2 Characteristics of included studies

The 14 studies included in the quantitative synthesis evaluated robotic and mechanized rehabilitation technologies as well as other non-pharmacological rehabilitation approaches (Fan et al., 2025; Huo et al., 2024; Liang et al., 2025; Tian et al., 2024; Kim et al., 2020; Wang et al., 2025; Li et al., 2025; Kim et al., 2025; Deng et al., 2021; Yu et al., 2025; Bei et al., 2023; Mao et al., 2022; Wang et al., 2024; Lee et al., 2025). The duration of interventions ranged from 2 to 8 weeks. Most studies were conducted in China and the Republic of Korea. FMA-LE data were available for the primary quantitative synthesis in all 14 studies. Detailed characteristics of the included studies are presented in Table 1.

**Table 1:** Characteristics of included randomized controlled trials (*n* = 14).

Study	Country	Study design and sample	Participants and stroke characteristics	Experimental intervention	Control intervention	Intervention dose and duration	Outcome measures
Fan et al. (2025)	China	Randomized controlled trial; 25 randomized, 22 analyzed (11/11)	Patients with first-ever stroke; 2 weeks to 6 months after stroke; predominantly subacute stage	Robotic-assisted gait training using the BEAR-H1 robotic exoskeleton in an assisted-walking mode, in addition to conventional rehabilitation	Conventional gait training with a therapist and conventional rehabilitation	30 min/session, twice daily, 5 days/week, for 4 weeks	Fugl-Meyer Assessment of the lower extremity; 6-Minute Walk Test; Functional Ambulation Category; gait parameters; cadence; electromyography and neuroplasticity-related measures
Huo et al. (2024)	China	Single-center, single-blind randomized controlled trial; 40 randomized, 30 analyzed (14/16)	Patients with first-ever unilateral supratentorial stroke; up to 6 months after stroke; subacute stage	Robotic-assisted overground gait training using the LiteStepper unilateral lower-limb exoskeleton, in addition to conventional rehabilitation	Conventional rehabilitation including joint mobilization, muscle strengthening, stretching, motor control, transfer, balance, and gait training	30 min/day, 5 days/week, for 4 weeks	Fugl-Meyer Assessment of the lower extremity; Berg Balance Scale; Modified Barthel Index; Functional Ambulation Category; gait parameters; functional near-infrared spectroscopy
Liang et al. (2025)	China	Randomized controlled trial; 46 randomized and analyzed (23/23)	Patients with first-ever stroke; 1–6 months after stroke; subacute stage; unilateral hemiparesis	Robotic-assisted gait training using the Kickstart Walk Assist portable lower-limb exoskeleton with Exotendon technology, in addition to standard rehabilitation	Standard rehabilitation and conventional gait exercises	20 min/session, 5 days/week, for 4 weeks; standard rehabilitation was provided for 40 min/day	Fugl-Meyer Assessment of the lower extremity; walking speed; step length; temporal symmetry; 10-Meter Walk Test; electromyography; additional gait parameters
Tian et al. (2024)	China	Four-arm randomized controlled trial; 100 patients, 25 per group. Lower-limb robot training versus conventional rehabilitation was used for the present meta-analysis	Patients with cerebral infarction or cerebral hemorrhage; 2 weeks to 6 months after stroke; recovery/subacute stage	Lower-limb robotic training using the AIWALKER robotic system, in addition to standard conventional rehabilitation	Standard conventional rehabilitation	20 min/session for 2 weeks; 5 days/week. The publication reports inconsistent information regarding once- versus twice-daily frequency	Fugl-Meyer Assessment, including the lower-extremity component; Berg Balance Scale; Barthel Index; Pittsburgh Sleep Quality Index; Fatigue Scale-14; Hamilton Anxiety Scale; Hamilton Depression Scale
Kim et al. (2020)	Republic of Korea	Randomized controlled trial; 30 randomized, 28 analyzed (14/14)	Patients approximately 3–12 months after stroke; late subacute/chronic stage	Robotic gait training using the G-EO System Evolution, in addition to conventional rehabilitation	Body-weight-supported treadmill gait training	30 min/day, 5 days/week, for 4 weeks; 20 sessions	Fugl-Meyer Assessment of the lower extremity; Timed Up and Go Test; 10-Meter Walk Test; cortical activity assessed using functional near-infrared spectroscopy
Wang et al. (2025)	China	Randomized controlled trial; 33 randomized, 27 analyzed (14/13)	Patients with ischemic or hemorrhagic stroke; approximately 1 month after stroke; early post-stroke stage	Split-belt treadmill gait training with different belt speeds, with the paretic limb placed on the slower belt	Split-belt treadmill gait training with equal belt speeds	24 min/session, 5 sessions/week, for 4 weeks; 20 sessions	Fugl-Meyer Assessment of the lower extremity; Berg Balance Scale; Wisconsin Gait Scale; Timed Up and Go Test; walking speed; step length; spatial and temporal gait symmetry
Li et al. (2025)	China	Randomized controlled trial; 42 randomized, 36 analyzed (18/18)	Patients with first-ever stroke; 1–6 months after stroke; subacute/early post-stroke stage	Robotic-assisted treadmill gait training using the GR-A1 robotic system	Conventional overground gait training	30 min/session, 5 sessions/week, for 4 weeks; 20 sessions	Fugl-Meyer Assessment of the lower extremity; Functional Ambulation Category; functional near-infrared spectroscopy; quantitative electroencephalography
Kim et al. (2025)	Republic of Korea	Randomized controlled trial; 48 randomized, 44 analyzed (22/22)	Patients more than 6 months after stroke; chronic stage; ischemic or hemorrhagic stroke	High-intensity interval training combined with robotic-assisted gait training using the Morning Walk robotic system	Conventional treadmill gait training	30 min/session, 3 sessions/week, for 8 weeks; 24 sessions	10-Meter Walk Test; Functional Ambulation Category; 2-Minute Walk Test; Berg Balance Scale; Fugl-Meyer Assessment of the lower extremity; maximal oxygen consumption; Modified Barthel Index
Deng et al. (2021)	China	Single-center, single-blind randomized controlled trial; 85 randomized, 54 analyzed (27/27)	Patients with first-ever middle cerebral artery stroke; less than 12 weeks after stroke; subacute/early post-stroke stage	Robotic-assisted gait training using the A3 Gait Training and Evaluation System, in addition to conventional physiotherapy	Conventional overground gait training with conventional physiotherapy	2 h/day for 14 consecutive days; robotic training was provided in two 50-min periods separated by a 20-min rest interval	Fugl-Meyer Assessment of the lower extremity; Timed Up and Go Test; spatiotemporal gait parameters; walking speed; step length; cadence; gait symmetry
Yu et al. (2025)	China	Three-arm randomized controlled trial; 45 analyzed, 15 per group	Patients with first-ever stroke; 3 weeks to 6 months after stroke; subacute/recovery stage	Active-assisted robotic lower-limb training plus standard rehabilitation	Standard rehabilitation without robotic training	20 min/day, 5 days/week, for 2 weeks; 10 sessions	Fugl-Meyer Assessment of the lower extremity; motor evoked potentials; functional near-infrared spectroscopy
Bei et al. (2023)	China	Two-arm randomized controlled trial; 160 patients, 80 per group	Patients with first-ever stroke; less than 1 month after stroke; early/acute stage	Water exercise therapy, including aquatic hemiplegia exercises and water-walking training, plus conventional rehabilitation	Conventional rehabilitation including spasticity-reduction exercises, standing training, neuromuscular facilitation, joint mobility exercises, lower-limb weight-bearing, and activities of daily living training	30–40 min/day, 6 days/week, for 8 weeks; 48 sessions	Fugl-Meyer Assessment of the lower extremity; Functional Ambulation Category; Berg Balance Scale; Modified Barthel Index; National Institutes of Health Stroke Scale; modified Rankin Scale
Mao et al. (2022)	China	Parallel-group randomized controlled trial; 32 randomized, 31 analyzed (16/15)	Patients with ischemic stroke; ≥12 months after stroke; chronic stage; post-stroke foot drop	Transcutaneous peroneal nerve stimulation during walking using the XFT-2001D stimulator	Conventional home/community gait training and motor relearning exercises without electrical stimulation	3 weeks; 15–60 min/day during week 1 and >60 min/day during weeks 2–3; approximately 2 h/day of total gait training in both groups	Fugl-Meyer Assessment of the lower extremity; Berg Balance Scale; spatiotemporal gait parameters; three-dimensional kinematic and kinetic parameters
Wang et al. (2024)	China	Randomized, double-blind pilot trial; 32 patients (16/16)	Patients with ischemic or hemorrhagic stroke; approximately 8.5 months after stroke; predominantly chronic stage	Real repetitive transcranial magnetic stimulation combined with visual-feedback cycling exercise	Sham repetitive transcranial magnetic stimulation combined with the same visual-feedback cycling exercise	5 sessions/week for 2 weeks; 10 sessions; cycling 20 min/session; repetitive transcranial magnetic stimulation for 15 min	Fugl-Meyer Assessment of the lower extremity; Berg Balance Scale; 2-Minute Walk Test; gait parameters; motor evoked potentials
Lee et al. (2025)	Republic of Korea	Randomized controlled trial; 33 patients (19/14)	Patients with stroke within the previous 24 months and impaired balance; exact stroke stage was not specified by the authors	Balance training using the Balance Pro SBT-330 device, incorporating weight shifting, visual feedback, and game-based exercises, plus conventional rehabilitation	Conventional rehabilitation group (CRG)—conventional physical therapy without additional training on the SBT-330. Conventional rehabilitation included lower-limb strengthening, functional training (ergometer/stepper), gait training, and static/dynamic balance training.	SBT-330 group: 30 min/day, 5 days/week for 4 weeks. Each session consisted of 15 min of fruit-catching game and 15 min of archery game. CRG: 30 min/day of conventional physical therapy, 5 days/week for 4 weeks.	Primary outcome: change in Berg Balance Scale (BBS) score. Secondary outcomes: Timed Up and Go (TUG), Fugl-Meyer Assessment of the Lower Extremity (FMA-LE), Functional Ambulation Category (FAC), Korean Modified Barthel Index (K-MBI), and Korean Trunk Impairment Scale (K-TIS).

### 3.3 Risk of bias assessment

Among the 14 randomized controlled trials included in the quantitative synthesis, 12 studies (85.7%) were judged to have some concerns regarding risk of bias, while two studies (14.3%) were judged to be at high overall risk of bias. No study was judged to have a low overall risk of bias.

The main concerns were related to the randomization process, deviations from intended interventions, missing outcome data, and selection of the reported result. In particular, insufficient information regarding allocation procedures and deviations from the intended interventions contributed to judgments of some concerns in several studies. Two studies were judged to have a high overall risk of bias, primarily because of concerns in multiple RoB 2 domains. Overall, the risk-of-bias assessment indicated that methodological limitations were present across the evidence base and were considered when interpreting the pooled results. The distribution of risk of bias across domains is presented in Figure 2.

**Figure 2:**
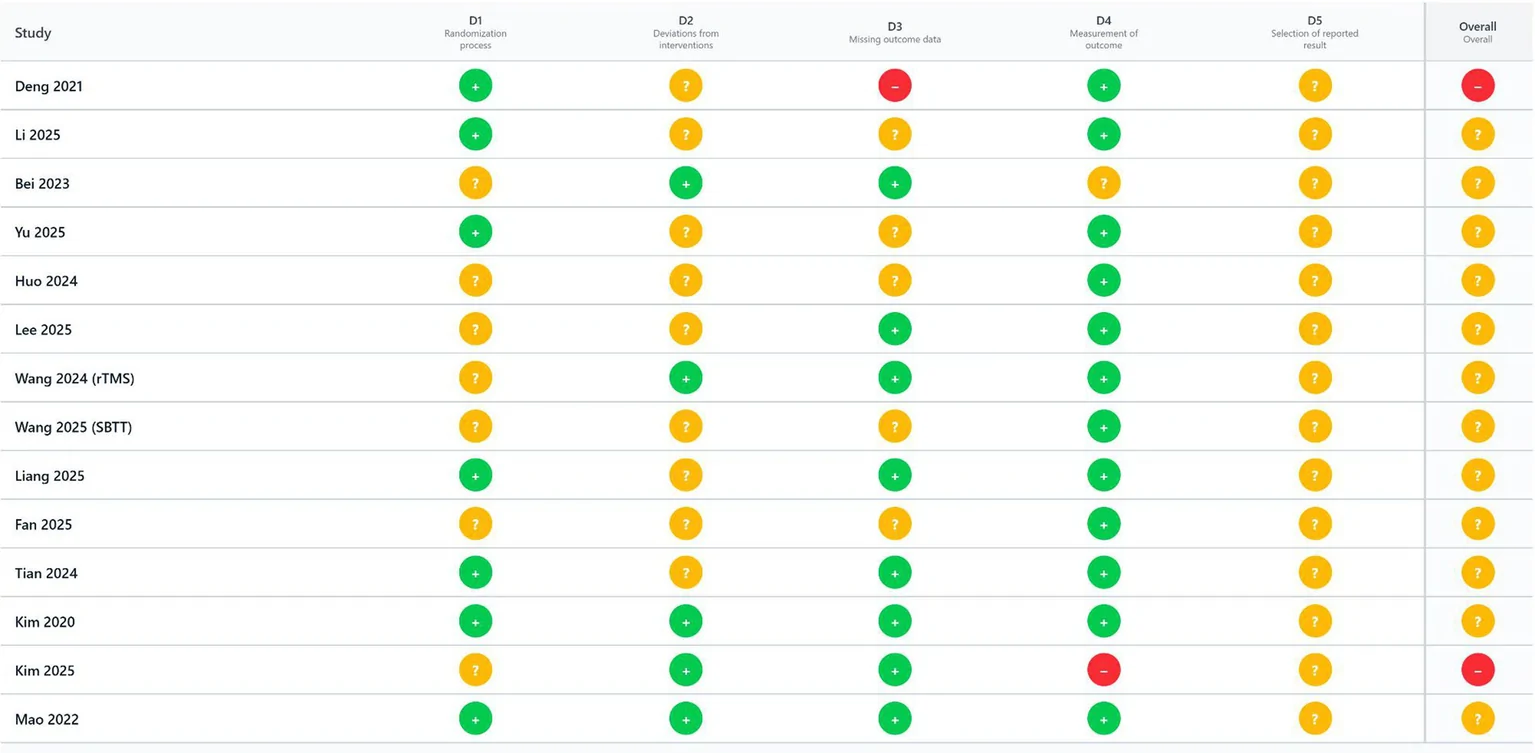
Risk of bias assessment of the 14 randomized controlled trials included in the quantitative synthesis using the Cochrane Risk of Bias 2 (RoB 2) tool. The figure summarizes judgments across the five risk-of-bias domains and the overall risk of bias for each included study.

### 3.4 Meta-analysis results

The overall effect of the included rehabilitation interventions on lower limb motor function is presented in Figure 3. A random-effects model demonstrated a statistically significant improvement in FMA-LE scores in the intervention groups compared with conventional rehabilitation (MD = 3.06; 95% CI 1.97–4.16; *p* < 0.001). The positive mean difference indicates higher post-intervention FMA-LE scores in participants receiving the investigated interventions.

**Figure 3:**
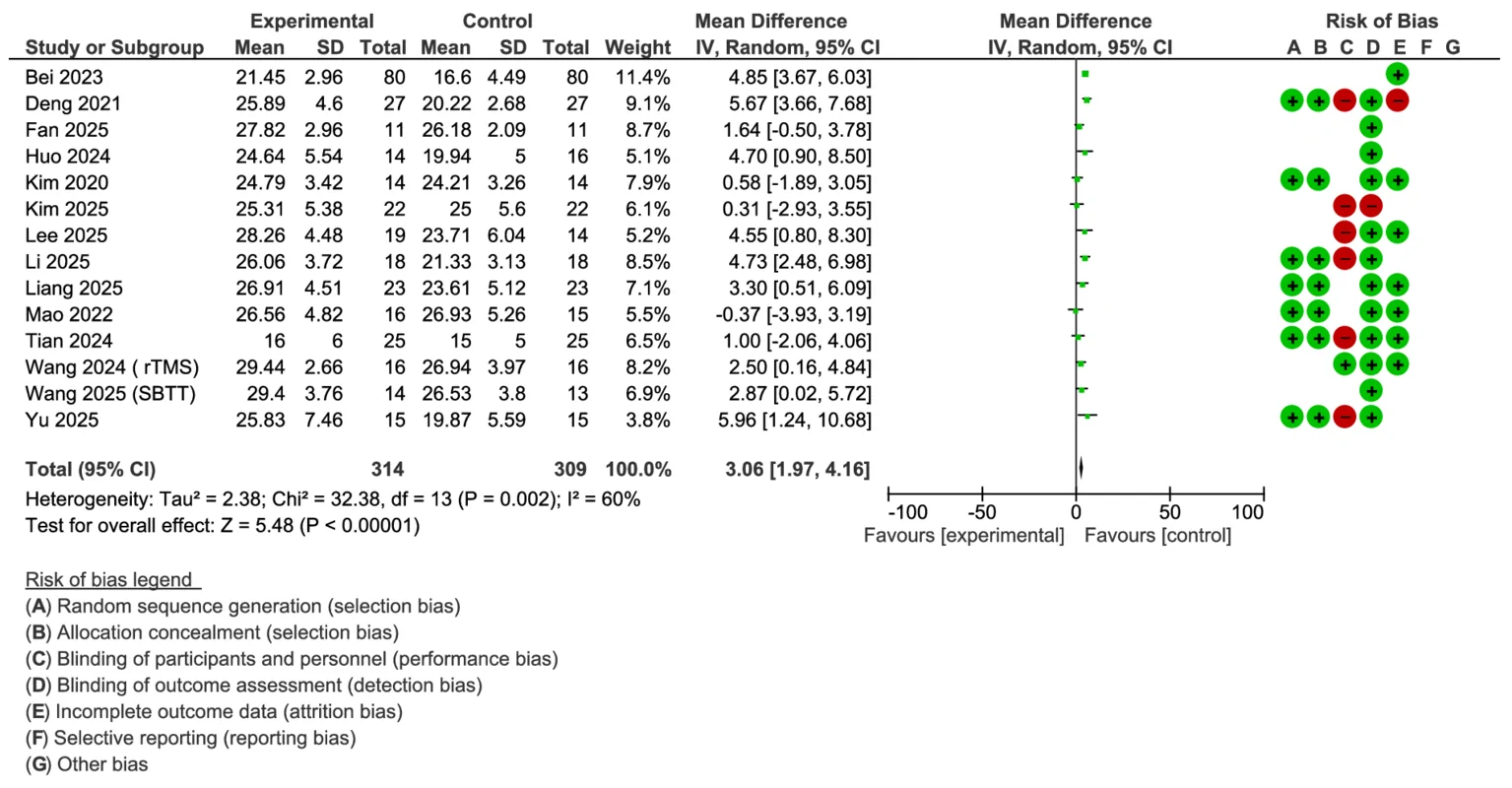
Forest plot of the effects of non-pharmacological rehabilitation interventions on lower limb motor function after stroke, measured by the Fugl–Meyer Assessment of the lower extremity (FMA-LE). Effects are presented as mean differences (MD) with 95% confidence intervals using a random-effects model. Subgroup analyses were performed according to intervention type.

Substantial between-study heterogeneity was observed (*I*^2^ = 59.8%), indicating variability in the magnitude of treatment effects across the included studies (Table 2).

**Table 2:** Post-intervention quantitative data for the primary outcome.

Study	Intervention group, *n*	FMA-LE, mean ± SD	Control group, *n*	FMA-LE, mean ± SD	Comparison included in the meta-analysis
Fan et al. (2025)	11	27.82 ± 2.96	11	26.18 ± 2.09	Robotic exoskeleton vs. conventional rehabilitation
Huo et al. (2024)	14	24.64 ± 5.54	16	19.94 ± 5.00	Unilateral exoskeleton vs. conventional rehabilitation
Liang et al. (2025)	23	26.91 ± 4.51	23	23.61 ± 5.12	Kickstart exoskeleton vs. conventional rehabilitation
Tian et al. (2024)	25	16.00 ± 6.00	25	15.00 ± 5.00	Lower-limb robotic training vs. conventional rehabilitation^*^
Kim et al. (2020)	14	24.79 ± 3.42	14	24.21 ± 3.26	End-effector robotic gait training vs. body-weight-supported treadmill training
Wang et al. (2025)	14	29.40 ± 3.76	13	26.53 ± 3.80	Split-belt treadmill training vs. conventional treadmill training
Li et al. (2025)	18	26.06 ± 3.72	18	21.33 ± 3.13	Robot-assisted gait training vs. conventional overground gait training
Kim et al. (2025)	22	25.31 ± 5.38	22	25.00 ± 5.60	High-intensity interval training with robotic gait therapy vs. treadmill gait training
Deng et al. (2021)	27	25.89 ± 4.60	27	20.22 ± 2.68	Robot-assisted gait training vs. conventional gait training
Yu et al. (2025)	15	25.83 ± 7.46	15	19.87 ± 5.59	Active-assisted robotic training vs. conventional rehabilitation^**^
Bei et al. (2023)	80	21.45 ± 2.96	80	16.60 ± 4.49	Water exercise therapy vs. conventional rehabilitation
Mao et al. (2022)	16	26.56 ± 4.82	15	26.93 ± 5.26	Foot-drop stimulation vs. conventional gait training
Wang et al. (2024)	16	29.44 ± 2.66	16	26.94 ± 3.97	Repetitive transcranial magnetic stimulation plus visual-feedback cycling vs. sham stimulation
Lee et al. (2025)	19	28.26 ± 4.48	14	23.71 ± 6.04	Balance training with Balance Pro SBT-330 vs. conventional rehabilitation

Total: 14 randomized controlled trials; 623 participants (314 in the intervention groups and 309 in the control groups) (Fan et al., 2025; Huo et al., 2024; Liang et al., 2025; Tian et al., 2024; Kim et al., 2020; Wang et al., 2025; Li et al., 2025; Kim et al., 2025; Deng et al., 2021; Yu et al., 2025; Bei et al., 2023; Mao et al., 2022; Wang et al., 2024; Lee et al., 2025).

Values represent post-intervention FMA-LE scores used in the primary quantitative synthesis.

a. Tian et al. (2024) included four groups (control, Daoyin, lower-limb robotic training, and combined Daoyin plus robotic training). Only the lower-limb robotic training versus control comparison was included in the primary analysis to avoid introducing the additional Daoyin component.

b. Yu et al. (2025) included three groups (control, passive robotic training, and active-assisted robotic training). The active-assisted versus control comparison was selected for the primary analysis; the passive-versus-control comparison was not included simultaneously because this would result in reuse of the same control group.

c. Park et al. (2021) was not included in this table because the reported outcome was a synergy subscore rather than the prespecified total FMA-LE score and therefore was not eligible for the primary quantitative synthesis.

A subgroup analysis was performed according to intervention type. Studies were categorized into robotic and mechanized rehabilitation technologies (n = 10) and other non-pharmacological rehabilitation approaches (n = 4). In the subgroup of robotic and mechanized technologies, a statistically significant effect in favor of the intervention was observed (MD = 3.00; 95% CI 1.66–4.33), with substantial heterogeneity (*I*^2^ = 57%). The subgroup of other non-pharmacological interventions also demonstrated a statistically significant improvement in FMA-LE scores (MD = 3.15; 95% CI 0.94–5.35), with substantial heterogeneity (*I*^2^ = 68%). There was no statistically significant difference between the two intervention subgroups (χ^2^ = 0.01, df = 1, *p* = 0.91; *I*^2^ = 0%). The results of the subgroup analysis are presented in Figure 4.

**Figure 4:**
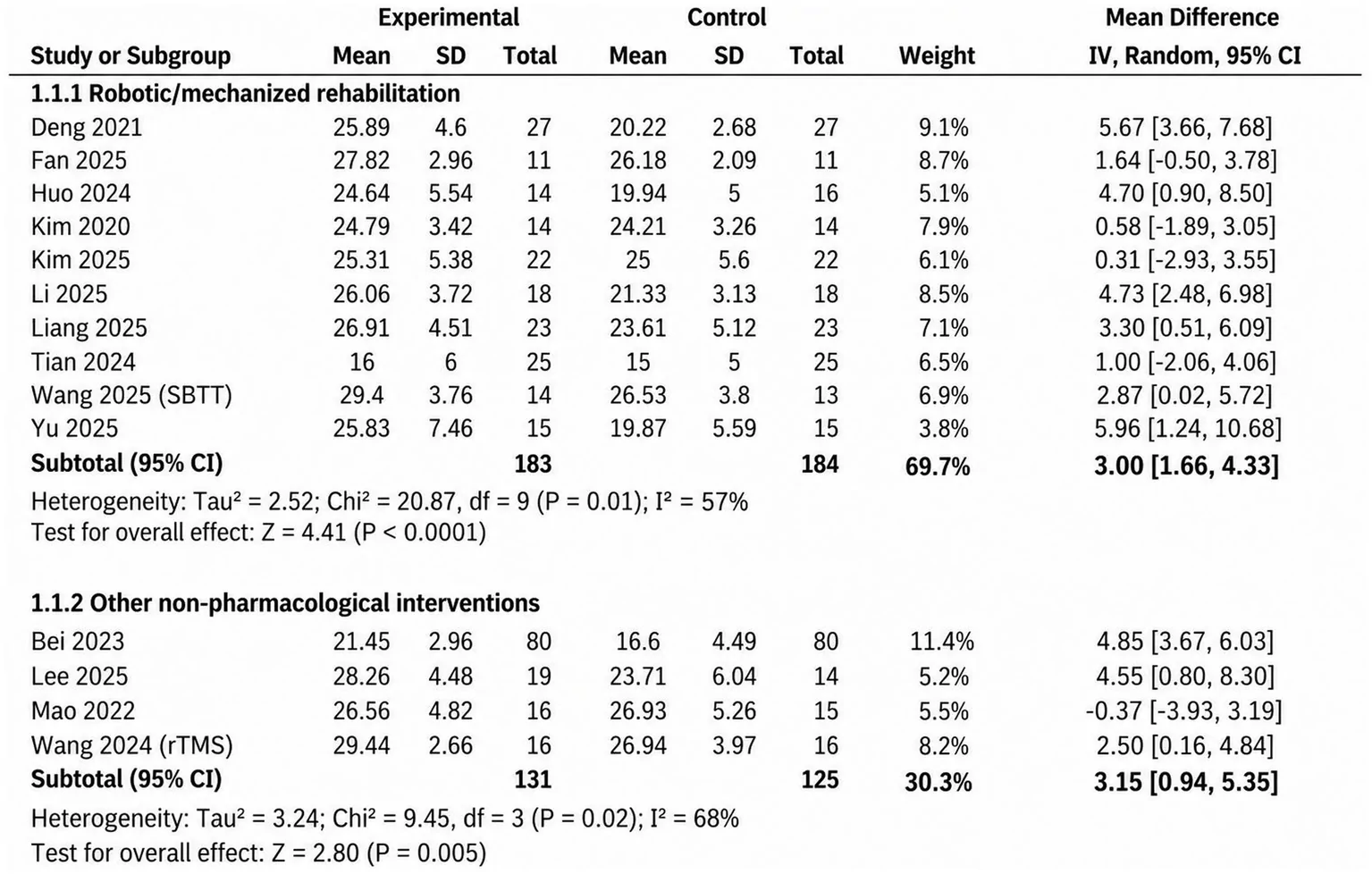
Subgroup analysis of the effects of non-pharmacological rehabilitation interventions on lower limb motor function after stroke, measured by the Fugl–Meyer Assessment of the lower extremity (FMA-LE). Effects are presented as mean differences (MD) with 95% confidence intervals using a random-effects model. Studies were grouped according to intervention type: Robotic and mechanized rehabilitation technologies and other non-pharmacological rehabilitation approaches.

### 3.5 Sensitivity analysis

A leave-one-out sensitivity analysis was performed to assess the robustness of the pooled estimate by sequentially excluding each of the 14 studies included in the quantitative synthesis. The pooled effect remained statistically significant and in favor of the intervention after exclusion of every individual study. The pooled MD ranged from 2.81 to 3.29, compared with 3.06 (95% CI 1.97–4.16) in the primary analysis. Between-study heterogeneity remained substantial, with *I*^2^ values ranging from 52 to 63% across the leave-one-out analyses.

These findings indicate that the overall effect was robust and was not substantially driven by any single study (Table 3).

**Table 3:** Leave-one-out sensitivity analysis of the meta-analysis.

Study omitted	Pooled mean difference (95% confidence interval)	*I*^2^ heterogeneity (%)
Bei et al. (2023)	2.83 (1.70–3.97)	52
Deng et al. (2021)	2.81 (1.69–3.92)	56
Fan et al. (2025)	3.20 (2.05–4.35)	59
Huo et al. (2024)	2.97 (1.82–4.12)	62
Kim et al. (2020)	3.29 (2.21–4.37)	55
Kim et al. (2025)	3.25 (2.15–4.34)	58
Lee et al. (2025)	2.98 (1.83–4.12)	63
Li et al. (2025)	2.90 (1.73–4.08)	61
Liang et al. (2025)	3.04 (1.86–4.21)	63
Mao et al. (2022)	3.27 (2.20–4.35)	57
Tian et al. (2024)	3.21 (2.09–4.33)	60
Wang et al. (2024) (repetitive transcranial magnetic stimulation)	3.10 (1.92–4.29)	62
Wang et al. (2025) (split-belt treadmill training)	3.07 (1.90–4.24)	63
Yu et al. (2025)	2.95 (1.82–4.07)	62

Values represent the pooled mean difference and corresponding 95% confidence interval after sequential exclusion of each individual study. *I*^2^ represents the percentage of variability across studies attributable to heterogeneity rather than sampling error. The primary meta-analysis included 14 randomized controlled trials.

### 3.6 Certainty of evidence

According to the GRADE approach, the overall certainty of evidence for the primary outcome (FMA-LE) was rated as moderate. The certainty of evidence was downgraded by one level for inconsistency because substantial statistical heterogeneity was observed across the included studies (*I*^2^ = 60%).

Although two studies were judged to have a high overall risk of bias, no downgrade was applied for risk of bias because these studies represented a limited proportion of the overall evidence, and leave-one-out sensitivity analyses showed that their individual exclusion did not materially alter the direction or statistical significance of the pooled effect (Table 4).

**Table 4:** Certainty of evidence assessed using the GRADE approach.

Outcome	Number of studies	Participants	Effect estimate	Risk of bias	Inconsistency	Indirectness	Imprecision	Other considerations	Certainty
Lower limb motor function (FMA-LE), post-intervention	14	623	MD 3.06 (1.97–4.16)	Not serious	Serious ^a^	Not serious	Not serious	Publication bias not formally assessed	Moderate ⨁⨁⨁◯

The certainty of evidence was assessed using the GRADE approach.

a. Substantial statistical heterogeneity was observed among the included studies (*I*^2^ = 60%; *χ*^2^ = 32.38, df = 13, *p* = 0.002), indicating variability in the magnitude of treatment effects. Therefore, the certainty of evidence was downgraded by one level for inconsistency.

## 4 Discussion

The present systematic review and meta-analysis demonstrated that non-pharmacological rehabilitation interventions are associated with a statistically significant improvement in lower limb motor function in adults after stroke, as assessed by the Fugl–Meyer Assessment for the lower extremity (FMA-LE). Across 14 randomized controlled trials included in the quantitative synthesis, the pooled effect favored the intervention groups over conventional rehabilitation (MD = 3.06; 95% CI 1.97–4.16). Substantial between-study heterogeneity was observed (*I*^2^ = 60%), indicating variability in the magnitude of treatment effects across studies.

The subgroup analysis showed statistically significant improvements in both intervention categories. Robotic and mechanized rehabilitation technologies demonstrated a pooled effect of MD = 3.00 (95% CI 1.66–4.33), while other non-pharmacological rehabilitation approaches demonstrated a pooled effect of MD = 3.15 (95% CI 0.94–5.35). However, the difference between the two subgroups was not statistically significant (χ^2^ = 0.01, df = 1, *p* = 0.91; *I*^2^ = 0%). Therefore, the present findings do not support the conclusion that robotic or mechanized technologies are superior to other non-pharmacological rehabilitation approaches. Rather, the results suggest that different rehabilitation strategies may provide beneficial effects on lower limb motor function when compared with conventional rehabilitation.

The observed benefits of robotic and mechanized rehabilitation may be related to their ability to provide repetitive, task-oriented and controlled motor training. Robotic systems can facilitate repeated gait-related movements while allowing adjustment of training intensity and the level of assistance according to patients’ functional abilities. Mechanized approaches, including treadmill-based and assisted gait training, may similarly provide structured and repetitive practice. These characteristics may contribute to motor relearning and functional recovery after stroke. The present findings are broadly consistent with previous systematic reviews and meta-analyses reporting beneficial effects of technology-assisted rehabilitation on post-stroke motor recovery (Amirbekova et al., 2025).

At the same time, the findings indicate that beneficial effects are not limited to robotic technologies. The subgroup of other non-pharmacological interventions, including water exercise therapy, peripheral stimulation, repetitive transcranial magnetic stimulation, and balance-oriented rehabilitation, also demonstrated a statistically significant pooled improvement in FMA-LE. The confidence interval in this subgroup was wider and heterogeneity was substantial (*I*^2^ = 68%), which may reflect differences in intervention mechanisms, treatment intensity, patient characteristics, and rehabilitation protocols. These findings support the use of a broader range of non-pharmacological rehabilitation strategies rather than focusing exclusively on robotic technologies.

The certainty of evidence for the primary outcome was rated as moderate according to the GRADE framework. The evidence was downgraded by one level for inconsistency because substantial statistical heterogeneity was observed across the included studies (*I*^2^ = 60%). Although two studies were judged to have a high overall risk of bias, their individual exclusion in leave-one-out sensitivity analyses did not materially change the direction or statistical significance of the pooled effect.

No downgrading was applied for indirectness or imprecision.

Several limitations should be considered when interpreting these findings. First, although the search was conducted in PubMed (MEDLINE), Scopus, and the Cochrane Library, the number of information sources was limited, and relevant studies indexed in other databases may have been missed. Second, several included trials had relatively small sample sizes, which may have contributed to variability in individual effect estimates. Third, the interventions were heterogeneous with respect to their mechanisms, training protocols, intensity, and duration, which was reflected in the substantial overall heterogeneity and higher heterogeneity within the subgroup of other interventions. Fourth, most studies evaluated relatively short-term post-intervention outcomes, limiting conclusions regarding the persistence of treatment effects over longer follow-up periods. Finally, one eligible randomized controlled trial, Park et al. (2021), was not included in the quantitative synthesis because it reported FMA-LE synergy subscores rather than the prespecified total FMA-LE outcome.

The sensitivity analysis provides additional support for the robustness of the findings. Sequential exclusion of each of the 14 studies did not eliminate the statistically significant positive effect, with pooled estimates ranging from MD = 2.81 to MD = 3.29. This indicates that the overall result was not substantially driven by any single study, including the largest trial.

These findings have potential clinical implications for post-stroke rehabilitation. Non-pharmacological interventions may be considered useful adjuncts to conventional rehabilitation for improving lower limb motor function. The absence of a statistically significant difference between the robotic/mechanized and other intervention subgroups suggests that treatment selection should not be based solely on the assumption that robotic technologies are superior. Factors such as patient characteristics, stage of recovery, availability of rehabilitation equipment, staff expertise, treatment intensity, feasibility, and resource requirements should also be considered when selecting an appropriate rehabilitation strategy.

Future research should focus on adequately powered, multicenter randomized controlled trials with standardized intervention protocols, clearly described randomization and allocation procedures, and longer follow-up periods. Future studies should also report clinically interpretable changes in FMA-LE scores and provide sufficient information to facilitate quantitative synthesis. Comparative studies directly evaluating different rehabilitation technologies and conventional approaches would be particularly valuable for determining which interventions are most effective for specific patient populations. In addition, evaluation of cost-effectiveness and feasibility may help determine how technology-assisted and other non-pharmacological rehabilitation approaches can be integrated into routine clinical practice.

## 5 Conclusion

This systematic review and meta-analysis demonstrated that non-pharmacological rehabilitation interventions targeting lower limb motor function after stroke are associated with a statistically significant improvement in FMA-LE scores compared with conventional rehabilitation. The pooled effect was MD = 3.06 (95% CI 1.97–4.16), with substantial between-study heterogeneity (*I*^2^ = 60%) and moderate certainty of evidence according to the GRADE framework.

Both robotic and mechanized rehabilitation technologies and other non-pharmacological rehabilitation approaches demonstrated statistically significant improvements in lower limb motor function. However, no statistically significant difference was observed between the intervention subgroups. These findings support the use of non-pharmacological rehabilitation as an adjunct to conventional rehabilitation after stroke, while the choice of intervention should consider patient characteristics, rehabilitation goals, available resources, and clinical feasibility.

## Data Availability

The data analyzed in this study were derived from previously published randomized controlled trials cited in the article. No original datasets were generated for this study.
